# Hemp inflorescence meal as a novel feed ingredient in laying hens: Safety assessment, nutritional characterization, and effects on egg quality

**DOI:** 10.14202/vetworld.2025.2406-2413

**Published:** 2025-08-21

**Authors:** Hathairat Saengsuwan, Chaiyapoom Bunchasak, Choawit Rakangthong, Kanokporn Poungpong

**Affiliations:** 1 Department of Animal Science, Faculty of Agriculture, Kasetsart University, Bangkok 10900, Thailand; 2 Department of Physiology, Faculty of Medicine, Kasetsart University, Bangkok 10900, Thailand

**Keywords:** cannabinoids, egg quality, feed safety, hemp byproducts, poultry nutrition, sustainable feed ingredient

## Abstract

**Background and Aim:**

Hemp inflorescence meal (HIM) is a byproduct of cannabinoid extraction and offers a sustainable, nutrient-rich alternative for animal feed. However, its application in poultry diets remains limited due to concerns regarding residual cannabinoid transfer to eggs. This study aimed to evaluate the nutritional composition, metabolizable energy, production performance, egg quality, and cannabinoid residue safety of HIM when included in laying hen diets.

**Materials and Methods:**

HIM was analyzed for proximate composition, amino acid profile, and cannabinoid content (cannabidiol [CBD] and tetrahydrocannabinol [THC]) using standard analytical methods and liquid chromatography-diode array detection. Apparent metabolizable energy (AME) was determined through prediction equations and *in vivo* assays using chromium oxide as a marker. A total of 108 Lohmann Brown laying hens (84 weeks old) were assigned to control and treatment groups (10% HIM inclusion) and fed for 4 weeks. Production parameters and egg quality were measured weekly. Eggs were analyzed for cannabinoid residues at the end of the study.

**Results:**

HIM contained 25% crude protein, 11.8% crude fiber, and had a predicted AME of 2,098 kcal/kg, closely matching the *in vivo* AME of 2,110 kcal/kg. Trace CBD (6.27 mg/kg) was present in HIM, while THC was undetectable; no cannabinoid residues were found in eggs. No significant differences (p > 0.05) were observed in production performance between groups. However, HIM inclusion significantly improved albumen height (7.82 mm vs. 6.76 mm, p = 0.02), Haugh unit (86.73 vs. 80.30, p = 0.03), and reduced yolk-to-albumen ratio (39.02 vs. 40.59, p = 0.04).

**Conclusion:**

The inclusion of 10% HIM in laying hen diets is safe, as evidenced by the absence of cannabinoid residues in eggs and stable production performance. HIM enhanced egg white quality, likely due to its high protein content and bioactive compounds. These findings support HIM as a functional, sustainable feed component in poultry nutrition. Future studies should focus on optimal inclusion levels, amino acid supplementation, and long-term impacts on egg quality and bird health.

## INTRODUCTION

Hemp inflorescence meal (HIM) is a byproduct of cannabinoid extraction from the flowers of hemp (*Cannabis sativa* L.). As the industrial and medicinal applications of hemp continue to grow, the utilization of such byproducts has gained increasing importance [1]. HIM has emerged as a potentially sustainable feed com-ponent due to its favorable nutritional composition [1]. However, concerns persist regarding the presence of residual cannabinoids, particularly tetrahydrocannabinol (THC) and cannabidiol (CBD), which could be transferred to animal-derived products and potentially pose risks to human health. As a result, regulatory restrictions are in place concerning the inclusion of HIM in animal feed formulations.

Hemp is differentiated from marijuana by its low THC content, defined as <0.3%, which ensures the absence of psychoactive effects [2]. Research on the dietary inclusion of hemp seeds and hemp seed oil in poultry has demonstrated that cannabinoid residues are not detectable in eggs or tissues. Moreover, these feed ingredients have shown benefits such as improved oxidative stability and enhanced fatty acid profiles in eggs [1, 3].

Despite growing interest in the use of hemp byproducts in animal nutrition, particularly hemp seeds and hemp seed oil, research on HIM remains limited. Existing studies have largely focused on the nutritional value and safety of hemp-derived products in terms of performance metrics and residue analysis in poultry and livestock [1, 3–6]. However, there is a notable lack of data specifically addressing HIM, which differs in composition from seeds and oil due to its origin from floral parts remaining after cannabinoid extraction. This raises questions about its metabolizable energy value, amino acid profile, and the extent to which residual cannabinoids may remain or transfer into animal products. In addition, the safety of HIM inclusion in poultry diets, especially concerning cann-abinoid residue carryover into eggs, has not been comprehensively assessed using validated detection methods. Regulatory agencies require clear evidence of non-detectable or legally acceptable cannabinoid levels in animal-derived products, yet such data for HIM-fed poultry are currently insufficient. Furthermore, the impact of HIM on egg quality parameters – beyond just production performance – has received minimal scientific attention.

In light of these gaps, the present study was designed to evaluate the safety and efficacy of HIM as a novel feed ingredient for laying hens. Specifically, the objectives were to (i) characterize the nutritional composition and amino acid profile of HIM; (ii) determine its apparent metabolizable energy (AME) both through predictive equations and *in vivo* analysis; (iii) assess its impact on laying hen production performance and detailed egg quality metrics; and (iv) quantify any cannabinoid (THC and CBD) residues in eggs using validated liquid chromatography-diode array detection (LC-DAD) methods. By integrating com-positional, performance, and safety data, this study aims to determine the feasibility of incorporating HIM into layer diets, contributing to sustainable feed devel-opment while ensuring food safety for consumers.

## MATERIALS AND METHODS

### Ethical approval

All experimental procedures were approved by the Institutional Animal Care and Use Committee of Kasetsart University, Thailand (Approval No. ACKU67-AGR-013) and were conducted in compliance with the ARRIVE guidelines.

### Study period and location

The study was conducted from June 2022 to December 2023 in Department of Animal Science, Faculty of Agriculture, Kasetsart University, Bangkok, Thailand.

### Experimental animals and housing

A total of 108 Lohmann Brown laying hens (84 weeks old) were randomly allocated into two gro-ups. Each group consisted of 6 replications and 9 hens in each. Birds were housed in a closed-environment facility equipped with an evaporative cooling system. Water was provided *ad libitum*, and feed was offered in mash form. The lighting regimen was maintained at 16 h of light and 8 h of darkness (16L:8D) throughout the study duration [7]. All hens were monitored daily for signs of abnormal behavior or adverse health effects.

### Experimental design and diet formulation

Diets were formulated according to the National Research Council’s recommendations to meet the nutri-tional requirements of Lohmann Brown-Classic hens (Table 1). The control group was fed a standard basal diet, while the treatment group received a diet in which 10% of the basal feed was replaced with HIM. The fee-ding trial lasted for 4 weeks.

**Table 1:** Nutrient composition of diets containing hemp inflorescence meal compared with the control diet.

Nutrient composition	Control diet	Diet with hemp inflorescence meal
Gross energy (kcal/kg)	3,254.80	3,283.90
Metabolizable energy (kcal/kg)	2,750.48	2,750.34
Moisture	11.95	11.55
Protein	17.00	17.00
Fat	5.28	5.19
Fiber	5.30	5.40
Ash	12.95	13.60
Minerals (%)		
Calcium	3.73	3.73
Phosphorus	0.60	0.59
Available phosphorus	0.37	0.37
Chloride	0.35	0.34
Amino acids (%)		
Lysine	0.90	0.90
Methionine	0.46	0.50
Methionine and cysteine	0.73	0.73
Tryptophan	0.20	0.20
Threonine	0.64	0.64

### Chemical composition analysis of HIM

Dried HIM was ground and subjected to proximate analysis for moisture, crude protein, crude fat, ash, crude fiber (CF), and nitrogen-free extract (NFE) using Association of Official Analytical Chemists (AOAC) standard procedures [8]. Gross energy (GE) was determined by bomb calorimetry. Calcium and phosphorus concentrations were measured using indu-ctively coupled plasma-optical emission spectrometry and spectrophotometry, respectively [9].

The amino acid composition was analyzed at Central Laboratory (Thailand) Co., Ltd., in compliance with Commission Directive 98/64/EC. Samples were hydrolyzed with hydrochloric acid for 23 h, and cystine and methionine were analyzed after performic acid-phenol oxidation. Amino acids were separated using ion-exchange chromatography and quantified through post-column ninhydrin derivatization at 570 nm (440 nm for proline). Detection and quantification limits were reported at 5 pmol and 10 pmol, respectively [10].

### Cannabinoid content determination in HIM

CBD and THC levels in HIM were measured using LC-DAD following AOAC Official Method^SM^ 2018.11. Cannabinoids were extracted using ethanol and qua-ntified using certified standards. The method demo-nstrated limits of quantification between 0.003% and 0.10% (w/w), spike recovery rates of 96.7%–101.3%, and relative standard deviations (SDs) ranging from 0.3%–4.8% (repeatability) and 1.1%–5.1% (intermediate precision) [11].

### AME evaluation

#### Predicted AME calculation

AME values were predicted using nutritional data from proximate analysis based on the following equation:

AME (kcal/kg) = (−37.5 × CF) + (49.1 × ADF) + (25.6 × Ash) + (0.75 × GE) + 1682

Where CF = Crude fiber, ADF = Acid detergent fiber, GE = Gross energy (kcal/kg) [12].

#### In vivo AME determination

For *in vivo* AME assessment, hens were fed diets supplemented with 1% chromium oxide and celite as indigestible markers. Feeding duration was tracked from initial ingestion until marker passage was confi-rmed via green feces. Once marker-containing feed was consumed, hens returned to the experimental diet. The remaining feed was weighed, and fecal trays were cleaned to eliminate contaminants. Fecal samples underwent proximate analysis, and AME was calculated as follows:

AME (kcal/kg) = [(feed intake × GEdiet) – (excreta output × GEexcreta)]/feed intake

Where GEdiet = Gross energy of the diet and GEexcreta = Gross energy of the excreta [13].

### Production performance assessment

Production parameters – including body weight gain, feed intake, feed conversion ratio, hen-day egg production, egg mass, and egg weight – were evaluated over the 4-week study. Data were recorded daily and summarized weekly.

### Egg quality evaluation

Three eggs per replication were randomly selected each week for quality evaluation. Parameters assessed included egg weight, shell breaking strength, yolk color, albumen height, Haugh unit (HU), shell thickness, and component weight ratios.

### Cannabinoid residue analysis in eggs

At the end of the trial, three eggs were randomly collected from each replication to determine cannabi- noid residues. Quantification of CBD and THC in egg matrices followed an in-house LC-DAD method based on AOAC Official Method^SM^ 2018.11. The method was validated for egg matrices, maintaining performance metrics equivalent to those used in HIM analysis, including accuracy, precision, and detection sensitivity [11].

### Statistical analysis

All data were expressed as mean ± SD. An unpaired t-test assuming unequal variances was performed to compare means between control and treatment groups. Statistical analyses were conducted using the Statistical Package for the Social Sciences software (Version 25.0, IBM Corp., Armonk, NY, USA), with a significance level set at p < 0.05. Each treatment group consisted of 54 hens, and three eggs per replication were sampled weekly for quality analysis.

## RESULTS AND DISCUSSION

### Cannabinoid analysis and safety assessment

HIM contained trace levels of CBD (6.27 mg/kg), and THC was not detected, both of which were below the legal threshold (THC <0.3%) for industrial hemp [2]. Notably, no traces of CBD or THC were found in the eggs from hens that were fed the HIM diet. This aligns with the findings of previous research on hemp-based feed ingredients [4–6]. These findings suggest that hens likely metabolize or excrete cannabinoids, which prevents them from accumulating in the eggs and keeps consumers safe. Hepatic metabolism through cytochrome P450 enzymes is likely to mediate this process [14]. These results align with studies on other livestock, reporting no cannabinoid residues in milk or tissues [15], reinforcing the safety profile of HIM.

### Nutritional composition of the HIM

The nutritional composition of HIM is presented in Table 2. The proximate analysis shows that the moisture content of the HIM is 6.6%, which is similar to that of other plant-based feed ingredients such as soybean meal (6%–12%) and sunflower meal (8%–10%) [16]. This level helps maintain stability during storage. As for crude protein, HIM comes in at 25%, which is notably higher than that of traditional fibrous feedstuffs, such as wheat bran, which has around 15%–17% crude protein. However, the protein content of HIM remains lower than that of soybean meal, which typically ranges from 44% to 48% [1]. Dry beans also contain 21%–25% protein, providing a useful comparative benchmark [17]. The protein and amino acid profile of HIM suggests its potential as a partial replacement for conventional protein sources.

**Table 2:** Nutritional components of hemp inflorescence meal.

Composition	Hemp inflorescence meal
Moisture (%)	6.6
Crude fat (%)	2.2
Ash (%)	19.4
Crude fiber (%)	11.8
Protein (%)	25.0
Neutral detergent fiber (%)	26.9
Acid detergent fiber (%)	25.3
Nitrogen-free extract (%)	34.8
Gross energy (kcal/kg)	3,464.0
Metabolizable energy (kcal/kg)	2,098.7

### Amino acid profile and comparative evaluation

The amino acid profile of HIM demonstrates a diverse range of essential and non-essential amino acids, suggesting that it could be a valuable protein source for poultry diets (Table 3). Aspartic acid (3.05%) and glutamic acid (1.81%) are the standout amino acids in HIM, playing crucial roles in metabolic functions such as energy production and neurotransmitter synthesis. Among the essential amino acids, leucine (1.07%) and arginine (1.08%), which are vital for muscle development and immune support in poultry, are present in notable quantities.

**Table 3:** Amino acid profiles of hemp inflorescence meal samples.

Amino acid (%)	Hemp inflorescence meal
Aspartic acid	3.05
Threonine	0.61
Serine	0.75
Glutamic acid	1.81
Glycine	0.93
Alanine	0.73
Cystine	-
Valine	0.89
Methionine	<0.20
Isoleucine	0.56
Leucine	1.07
Tyrosine	0.46
Phenylalanine	0.69
Histidine	0.17
Hydroxylysine	-
Lysine	0.81
Arginine	1.08
Hydroxyproline	<0.50
Proline	0.61
Tryptophan	<0.15

However, the lower levels of essential amino acids, such as methionine (<0.20%) and lysine (0.81%), might restrict the overall protein quality of HIM for poultry nutrition. In comparison to soybean meal, which is often regarded as the gold standard for plant-based protein in poultry diets due to its higher lysine (2.9%–3.1%) and methionine (0.6%–0.7%) contents [16], HIM has a considerably lower lysine content, highlighting the need for supplementation when it is used as a primary protein source.

Similarly, canola meal offers moderate amounts of lysine (approximately 2.0%–2.2%) and methionine (about 0.7%–0.8%) [18]. On the other hand, HIM has a notably lower methionine content, which might limit its use unless supplemented. However, HIM boasts a higher arginine content than canola meal, at approximately 1.08%. When compared to sunflower meal, which has about 1.5% lysine and 0.6% methionine [16], HIM’s lower lysine content could be a disadvantage, though its higher arginine level may provide a nutritional benefit in specific formulations.

Overall, while HIM’s amino acid profile indicates it could be a viable alternative protein source for poultry diets, its lower methionine and moderate lysine levels could restrict its effectiveness as a main protein source without additional fortification. Further studies are necessary to investigate the digestibility of the HIM amino acids.

### Crude nutrient composition and fiber analysis

The levels of crude fat (2.2%) and ash (19.4%) in HIM were notable. The ash content far exceeds that of soybean meal (6%–7%) and corn (1.5%) [16], likely due to residual mineral-rich floral structures in the inflor-escence. The high mineral content in this feed may red-uce the need for the addition of inorganic minerals to poultry diets.

The CF level is 11.8%, with neutral detergent fiber (NDF) at 26.9% and acid detergent fiber (ADF) at 25.3%, showing that HIM is rich in structural carbohydrates. Although these values are higher than those of soy-bean meal (NDF: 12%–15%, ADF: 8%–10%) [19], they are comparable to those of other agro-industrial byproducts, such as rice bran (NDF: 25%–30%) [20], suggesting that HIM could serve as a moderate-fiber feed ingredient.

The NFE content was 34.8%, indicating digestible carbohydrates. Although this number is lower than the NFE of corn, which typically ranges from 70% to 75%, it closely resembles that of alfalfa meal, which falls between 35% and 40% [16].

### AME estimation

The substitution of the proximate analysis values for HIM yielded a predicted AME of 2,098 kcal/kg. *In vivo* feeding trials with 84-week-old laying hens measured an AME of 2,110 kcal/kg. The equation used is specifically to predict poultry AME from proximate analysis data by inserting the measured values for CF, ADF, ash, and GE of a fibrous byproduct into the formula; one obtains its estimated AME – a difference of only 0.6% – demonstrating excellent agreement between prediction and observation.

HIM has higher quantities of CF (11.8%) and ADF (25.3%) than conventional poultry feed components; however, their negative influence on available energy is adequately accounted for by the prediction model. The slight increase in observed AME might be attributed to age-related digestive adaptations in older chickens or differences in fiber fermentability that are not fully represented by linear coefficients.

Future research should assess the energy contri-bution of HIM across different bird ages, gut-health conditions, and additional compositional factors, such as non-fiber carbohydrates or protein digestibility, to further refine the predictive model. The GE of HIM is 3,464 kcal/kg, and its predicted AME is 2,098 kcal/kg, both of which are comparable to high-fiber ingredients, such as wheat middlings, which have an AME range of 2,200–2,400 kcal/kg [21]. HIM is a moderate-energy feed ingredient. Hemp seeds boast an AME value of 4,308 kcal/kg [22].

### Comparison with other hemp byproducts

HIM exhibits intermediate nutritional value com-pared to other hemp byproducts. It has a protein content of 25%, which is greater than the 10%–15% in hemp hulls [1] but lower than the 30%–35% in hemp seed meal [23]. As a result, HIM falls within the category of intermediate protein sources.

It is a better choice for poultry diets because its CF level of 11.8% is far lower than the 40%–50% found in hemp hulls [5]. The comparatively high quantities of (ADF: 25.3%) and (NDF: 26.9%) indicate that the fiber component in HIM may restrict its inclusion rate in monogastric animals due to reduced digestibility. These characteristics underscore the potential of HIM as a viable feed ingredient, but they also highlight the importance of its proper formulation to maximize its effectiveness in poultry diets.

### Production performance evaluation

Hen-day egg production, feed intake, feed conv-ersion ratio, egg mass, and egg weight did not differ significantly (p > 0.05) between groups (Table 4). These results are consistent with those of prior studies eval-uating the application of hemp-derived products in pou-ltry diets.

**Table 4:** Effect of hemp inflorescence meal replacement in laying hen feed on production performance.

Parameter	Treatment		p-value

	Control	HIM replaced	
Hen-day egg production (%)	88.27 ± 6.76	86.16 ± 4.53	0.27
Feed intake (g/hen/day)	134.60 ± 0.79	134.33 ± 0.93	0.30
Feed conversion rate	2.48 ± 0.22	2.54 ± 0.11	0.28
Egg mass (g)	55.81 ± 4.60	54.13 ± 2.41	0.22
Egg weight (g)	63.21 ± 1.64	62.80 ± 1.19	0.32

HIM=Hemp inflorescence meal

Thus, when hemp seed oil was added to laying hen diets, Gakhar *et al*. [1] found no adverse effects on production performance. Further confirming the neu-tral effect of hemp-based ingredients on production performance, Neijat *et al*. [6] found no discernible changes in production performance when up to 30% hemp seeds and 9% hemp seed oil were added to the diet.

The high fat content in hemp-derived products, such as hemp seeds and hemp seed oil, is thought to influence production performance. Grobas *et al*. [24] noted that dietary fat levels can modulate energy use and egg production in laying hens as they age.

The absence of significant differences in prod-uction performance with HIM replacement may be partly attributed to its moderate fat content (2.2%), which provides a balanced energy source without disrupting metabolic processes. This observation aligns with that of Neijat *et al*. [6], who reported that the inclusion of hemp seed oil (up to 9%) did not negatively impact egg production or feed efficiency. The results indicate that HIM can be used as a replacement ingredient in laying hen diets without adversely affecting production performance.

### Egg quality analysis

The replacement of HIM in laying hen diets resu-lted in significant improvements in certain egg quality parameters, particularly albumen height and HUs, as shown in Table 5.

**Table 5:** Effect of hemp inflorescence meal replacement in laying hens on egg quality.

Parameter	Treatment		p-value

	Control	HIM replaced	
Egg weight (g)	63.21 ± 1.64	62.80 ± 1.19	0.32
Shell breaking strength (N)	37.46 ± 2.79	38.16 ± 3.26	0.35
Yolk color	6.82 ± 0.37	7.02 ± 0.14	0.13
Albumen height (mm)	6.76 ± 1.04	7.82 ± 0.29*	0.02
Haugh units	80.30 ± 6.93	86.73 ± 3.04*	0.03
Yolk weight ratio (%)	28.87 ± 0.43	28.29 ± 0.83	0.08
Albumen weight ratio (%)	71.13 ± 0.43	72.56 ± 2.05	0.06
Shell weight ratio (%)	11.42 ± 0.37	11.48 ± 0.72	0.43
Yolk: albumen ratio	40.59 ± 0.87	39.02 ± 1.72*	0.04
Shell thickness (mm)	0.90 ± 0.04	0.94 ± 0.03	0.09

* Significant differences with *P<*0.05. HIM=Hemp inflorescence meal

In comparison to the control group, hens given the HIM-replaced diet had greater albumen height (6.76 ± 1.04 mm vs. 7.82 ± 0.29 mm; p = 0.02) and HUs (80.30 ± 6.93 vs. 86.73 ± 3.04; p = 0.03). These findings indicate that dietary HIM replacement may enhance egg white quality, possibly due to its amino acid profile and bioactive compounds.

The HU, a widely recognized standard for assessing egg white quality, is strongly correlated with albumen height and egg weight [25]. These improvements may be attributed to the balanced amino acid profile of HIM, which likely promoted ovi-duct protein synthesis. Essential egg white proteins, such as ovalbumin, ovotransferrin, ovomucoid, and lysozyme, are generated in the tubular gland of the oviduct and play an important role in egg white deve-lopment [26].

As a novel feed ingredient, HIM has a high protein content of 25%, potentially enhancing its digestibility and bioavailability relative to conventional diets. The enhanced availability of HIM-derived dietary protein likely facilitated the synthesis of egg white proteins, thus improving albumen quality. The findings indicate that HIM serves as a viable protein source in the diets of laying hens, enhancing egg quality while maintaining safety standards.

### Bioactive components and functional effects

CBD, a non-psychoactive cannabinoid with several medicinal properties, is prevalent in hemp inflores-cences [27]. According to this study, HIM contains trace amounts of CBD (6.27 mg/kg). Several animal studies have shown that CBD has immunomodulatory, anxiolytic, anti-inflammatory, and antioxidant effects [28]. These attributes may benefit laying hens by decreasing stress, increasing immunity, and minimizing oxidative stress, all of which can affect egg quality.

Hemp has several bioactive components, inclu-ding flavonoids, terpenes, and phenolic compounds, which contribute to its antioxidant properties [29]. Antioxidants can help protect egg components by limi-ting lipid peroxidation. In addition, HIM likely contains a significant quantity of dietary fiber, which has been shown to be important for preserving poultry gut health by enhancing intestinal shape and supporting beneficial bacteria populations, both of which improve nutrient absorption [29]. Improved nutrient absorption ensures that the hens have sufficient access to all the nutrients required for egg production. In addition, the fiber content can contribute by increasing the overall digestive efficiency of the hens.

### Neutral impact on other egg traits

In contrast, no significant differences (p > 0.05) in egg weight, eggshell strength, yolk color, yolk weight ratio, albumen weight ratio, shell weight ratio, or shell thickness were observed between the control and HIM-replaced groups. These findings are in agreement with those reported by Silversides and Lefrancois [30], who reported that the inclusion of hemp seed meal (up to 20%) and hemp seed oil (up to 12%) in laying hen diets did not affect the weight of yolk, albumen, or shell, nor did it influence eggshell thickness or specific gravity.

Similarly, Neijat *et al*. [6] found that the inclusion of hemp seeds and hemp seed oil in poultry diets had no significant impact on eggshell quality or yolk color, further supporting the neutral effect of hemp-derived ingredients on these parameters. The lack of significant differences in yolk color suggests that HIM does not contain pigments that significantly alter yolk coloration, which is primarily influenced by carotenoid intake [31].

This finding is consistent with the low-fat content (2.2%) of HIM, which limits its contribution to lipid-soluble pigments. However, the significant reduction in the yolk-to-albumen ratio (40.59 ± 0.87 vs. 39.02 ± 1.72; p = 0.04) in the HIM-replaced group indicates a shift in the proportion of egg components, possibly due to improved protein utilization for albumen synthesis.

The observed improvements in albumen height and HUs following HIM replacement are comparable to the findings of studies involving other high-protein feed ingredients. For example, soybean meal, a widely used protein source in poultry diets, has been shown to enhance albumen quality due to its balanced amino acid profile [30]. Similarly, the inclusion of canola meal in laying hen diets has been reported to improve egg white quality without adversely affecting eggshell strength or yolk color [18]. These results suggest that HIM, similar to other plant-based protein sources, can positively influence egg quality when included in balanced diets.

## CONCLUSION

This study establishes HIM as a safe, nutritionally valuable, and functionally beneficial feed ingredient for laying hens. The HIM used in the trial contained 25% crude protein, 11.8% CF, and demonstrated a predicted AME of 2,098 kcal/kg, which was closely confirmed by *in vivo* measurements (2,110kcal/kg). No THC was detected, and CBD was present in trace amounts (6.27 mg/kg), both below legal limits. Crucially, cannabinoid residues were not found in any eggs, confirming the safety of HIM inclusion at 10% in poultry diets.

From a production standpoint, no adverse effects were observed on hen-day egg production, feed intake, feed conversion ratio, egg weight, or egg mass. Notably, HIM replacement significantly improved internal egg quality, with increased albumen height and HU values (p < 0.05), and a lower yolk-to-albumen ratio, indicative of enhanced protein utilization and albumen synthesis. These improvements are likely due to the high-quality protein, amino acid diversity, and bioactive compounds inherent in HIM.

Practical implications of these findings include the valorization of HIM, an agricultural byproduct, as a sustainable alternative protein source that enhances egg quality without compromising safety or productivity. This could reduce feed costs, lower reliance on conventional protein sources like soybean meal, and promote circular bioeconomy practices within the poultry industry.

Strengths of the study include comprehensive nutritional characterization, validated analytical met-hods for cannabinoid quantification, and both predicted and empirical AME assessments. In addition, the study employed a standard commercial layer strain at a late laying stage (84 weeks), increasing the relevance of the findings for industry application.

However, a key limitation is the short trial duration (4 weeks) and the fixed inclusion rate of 10%, which may not capture potential cumulative effects, variability across life stages, or long-term impacts on health and egg production.

Future research should focus on evaluating graded inclusion levels of HIM, exploring its effects across different poultry breeds and age groups, and investigating potential synergies with amino acid supp-lementation or other functional feed additives. Long-term studies are necessary to assess safety during chronic exposure, reproductive performance, and imp-acts on immune and gut health.

HIM is a promising, safe, and nutritionally viable feed ingredient that not only sustains performance but also enhances egg quality in laying hens. Its incorporation into poultry diets aligns with the goals of sustainable livestock production and feed diversification, while maintaining food safety for consumers.

## AUTHORS’ CONTRIBUTIONS

HS: Collected samples and performed experiments. CB and CR: Provided technical assistance during the experiments. KP: Designed the experiments, collected samples, and revised the manuscript. All authors have read and approved the final version of the manuscript.
